# Splicing and expression dynamics of SR genes in hot pepper (*Capsicum annuum*): regulatory diversity and conservation under stress

**DOI:** 10.3389/fpls.2024.1524163

**Published:** 2025-01-23

**Authors:** Lin Li, Yueqin Zhang, Rui Zhang, Xiangtao Cen, Yongxiang Huang, Hanqiao Hu, Xingyu Jiang, Yu Ling

**Affiliations:** ^1^ College of Coastal Agricultural Sciences, Guangdong Ocean University, Zhanjiang, China; ^2^ South China Branch of National Saline-Alkali Tolerant Rice Technology Innovation Center, Zhanjiang, China; ^3^ College of Agriculture and Food Engineering, Baise University, Baise, Guangxi, China

**Keywords:** hot pepper, alternative splicing (AS), serine/arginine-rich protein, abiotic stress, cross-species comparison

## Abstract

In this study, we identified and characterized 23 genes encoding serine/arginine-rich (SR) protein in hot pepper (*Capsicum annuum*), named CaSR here. These CaSR proteins are grouped into seven subfamilies. Phylogenetic analysis revealed a high degree of similarity between CaSRs and their homologous proteins in other plants. Promoter regions of SR proteins are enriched with elements relating to light response, stress, hormone signaling, and plant growth. Notably, transcription levels of several proteins, including CaSR33, CaSR34, and CaSR34a, were upregulated by salt, drought, and cold stresses, suggesting potential roles of these proteins in stress tolerance. We also observed an increase of CaSR transcript population resulting from alternative splicing (AS) regulation, mainly intron retention. AS patterns of CaSR genes varied among tissues. Higher AS intensity was found in the RS subfamily, while some genes in the RSZ subfamily showed no AS regulation under the conditions used here. Interestingly, a cross-species comparative study with Arabidopsis (*Arabidopsis thaliana*) and tomato (*Solanum lycopersicum*) showed that many AS events impact the region which codes the RNA recognition motif (RRM) domain of the protein, indicating a conserved regulatory mechanism of SR proteins in plants. Our findings reveal the functional diversity and evolutionary conservation of SR proteins in hot pepper and highlight AS as a mechanism enhancing plant adaptability, providing insights for future stress-resistant crop development.

## 1 Introduction

Plants tightly control their gene expression patterns through a complex molecular mechanism at different layers (Duque, 2011; Zhu, 2016; Chen et al., 2024). Alternative splicing (AS) is a widespread posttranscriptional regulation in higher eukaryotes. Individual mRNA precursors undergo AS to produce different mRNA isoforms (Li et al., 2020). Therefore, AS is the most important contributor to the diversity of transcriptome and proteome during plant development and environment adaptation (Laloum et al., 2023; Tognacca et al., 2023; Ma et al., 2024). Up to 95% of genes in human cells are regulated by AS, and also, it has been reported that approximately 70% of multiexon genes in plants are regulated by AS (Marquez et al., 2012; Chamala et al., 2015). Numerous studies have shown that plants can use AS to fine-tune their physiology and metabolism under normal and stress conditions to maintain a balance between carbon fixation and resource allocation (Posé et al., 2013; Mastrangelo et al., 2012; Jiang et al., 2017). It is well known that common types of AS include exon jumping (ES), intron retention (IR), mutually exclusive exons (MXE), exon splicing (EIS), and alternative 5′ splice site (5′SS) events and 3′SS (Zhu et al., 2017; Kim et al., 2007).

The splicing of pre-mRNA is processed in the spliceosome, where AS occurs when splice sites are occupied differentially (Jabre et al., 2019). The spliceosome is a large ribonucleoprotein complex consisting of five snRNPs (U1, U2, U4, U5, and U6) and numerous proteins (Will and Lührmann, 2011; Wahl et al., 2009). Serine/arginine (SR)-rich proteins are a class of important splicing factors that regulate splicing site selection by binding to splicing enhancers on precursor mRNA and play a key role in pre-mRNA splicing regulation (Duque, 2011; Zahler et al., 1992). The N-terminal of SR protein contains one or two RNA-binding domains (RRMs) that are responsible for recognizing and binding specific RNA regions, while the C-terminal contains arginine/serine-rich (RS) domains that contribute to protein–protein interactions (Wu and Maniatis, 1993; Shepard and Hertel, 2009; Rosenkranz et al., 2021; Rauch et al., 2014).

Plant genomes contained more SR genes generally, when compared with those of animals. For example, only nine SR proteins have been found in humans (McFarlane and Graham, 2010); however, a total of 19 and 24 SR proteins have been identified in *Arabidopsis* and sweet potato, respectively. Interestingly, 15 and 18 SR genes from *Arabidopsis* and sweet potato undergo AS under stress, resulting in a significant increase of transcript diversity of SR genes (Palusa et al., 2007; Chen et al., 2022). The plant SR protein family can be further divided into seven subfamilies, namely, SR, RSZ, SCL, RS, RSZ, SR45, SC, and RS2Z, based on their sequence features (Richardson et al., 2011; Barta et al., 2010). Among these, SCL, RS2Z, and RS are plant-specific subfamilies, while the other subfamily members can be found in both plants and mammalian cells. Accordingly, it is suggested recently that some SR proteins may have evolved for plant-specific functions (Kumar et al., 2023).

Accumulating studies have shown that SR plays an important role in response to stress, hormone signaling, and plant development (Reddy and Shad Ali, 2011; Long and Caceres, 2009; Liu et al., 2024). For instance, in terms of stress and hormone signaling, the splicing factor AtSR45 regulates *Arabidopsis* salt stress tolerance in a splicing variant-dependent manner. Overexpression of the splicing variant SR45.1 partially released the inhibition effect caused by ABA (Xing et al., 2015). Correspondingly, a similar phenomenon has been found in a recent study (Albuquerque-Martins et al., 2023). Furthermore, SR45.1 was proved to be critical for salt tolerance (Albaqami et al., 2019). *Arabidopsis* with SCL30a gene knockout showed hypersensitivity to ABA and salt stress during seed germination, while overexpression of this gene reduced the hypersensitivity (Laloum et al., 2023). SR proteins may regulate plant growth and development through pre-mRNA splicing controls of itself and other genes. Overexpression of *Arabidopsis* SR protein AtSRp30 would lead to changes in AS variation of pre-mRNAs transcribed from its own coding gene (*AtSRp30*) and several other genes. Moreover, the AtSRp30-overexpressing plants show growth deficiency phenotypes, such as late flowering and enlarged flowers and rosette leaves (Lopato et al., 1999). Similarly, overexpression of another Arabidopsis SR protein, AtRSZ33, resulted in changes in splicing patterns of pre-mRNAs of *AtRSZ33* other genes. Thus, overaccumulation of this SR protein caused excessive cell division and expansion in the apex meristem to form multileaf primordium but, in contrast, inhibit cell division in the root meristem (Kalyna et al., 2003). Moreover, the *Arabidopsis* mutant with SR45 mutation (*atsr45-1*) showed different phenotypes, such as narrow leaves, prolonged flowering time, and changes in the number of petals and stamens (Ali et al., 2007).

Hot pepper, belonging to the Solanaceae family, is a popular and important cash crop. While SR proteins have been characterized in many organisms, no SR protein had previously been identified at the genomic level in hot pepper. In this study, we identified a total of 23 SR family genes from the published hot pepper genome. Moreover, we analyzed their physicochemical properties, phylogenetic relationships, cis-acting elements, gene structures, conserved motifs, and chromosomal distributions using bioinformatics methods. Additionally, we examined the tolerance of hot pepper seedlings to abiotic stresses, together with expressional regulation of CaSR genes in responding to these stress conditions. Specially, AS regulation of several CaSR genes was comprehensively compared with their homologous genes in tomato and *Arabidopsis* to evaluate the evolution characteristics of splicing regulation of SR genes in different plants.

## 2 Results

### 2.1 Identification, physicochemical properties, and chromosomal localization of SR genes in hot pepper

Based on the defining characteristics of SR proteins, which include an RNA recognition motif (RRM) and an arginine/serine-rich (RS) domain, we identified a total of 23 SR proteins (CaSRs) from the hot pepper genome, by using SR protein sequences from Arabidopsis and tomato as queries. Analysis of the physicochemical properties of these 23 predicted SR proteins (**Supplementary Table 1**) revealed that their lengths range from 160 amino acids (CaRSZ21a) to 457 amino acids (CaRS42). The protein sequences of CaRS41 and CaRSZ21a contain several undefined consecutive amino acids, which prevented accurate prediction of their molecular weight (MW) and isoelectric point (pI). For the remaining 21 CaSR proteins, MW values range from 19,550.53 Da (CaRSZ22) to 51,806.84 Da (CaRS42). Notably, these 21 SR proteins all exhibited high pI values, ranging from 5.93 (CaRSZ22) to 12.26 (CaSR45). Except for CaRSZ22, which is a weakly acidic protein, the other 20 SR proteins are basic. The average hydropathy index (GRAVY) for CaSR proteins ranges from −1.603 (CaSCL33) to 0.16 (CaRSZ22), with CaRSZ22 being hydrophobic, while the remaining 22 SR proteins are hydrophilic. Subcellular localization predictions using Cell-PLoc 2.0 suggested that nine CaSRs are localized to the nucleus, 12 are located in both the nucleus and chloroplast, and CaRSZ32 is found in both the nucleus and cytoplasm. CaSR41, however, was predicted to be localized exclusively in the chloroplast.

**Figure 1:**
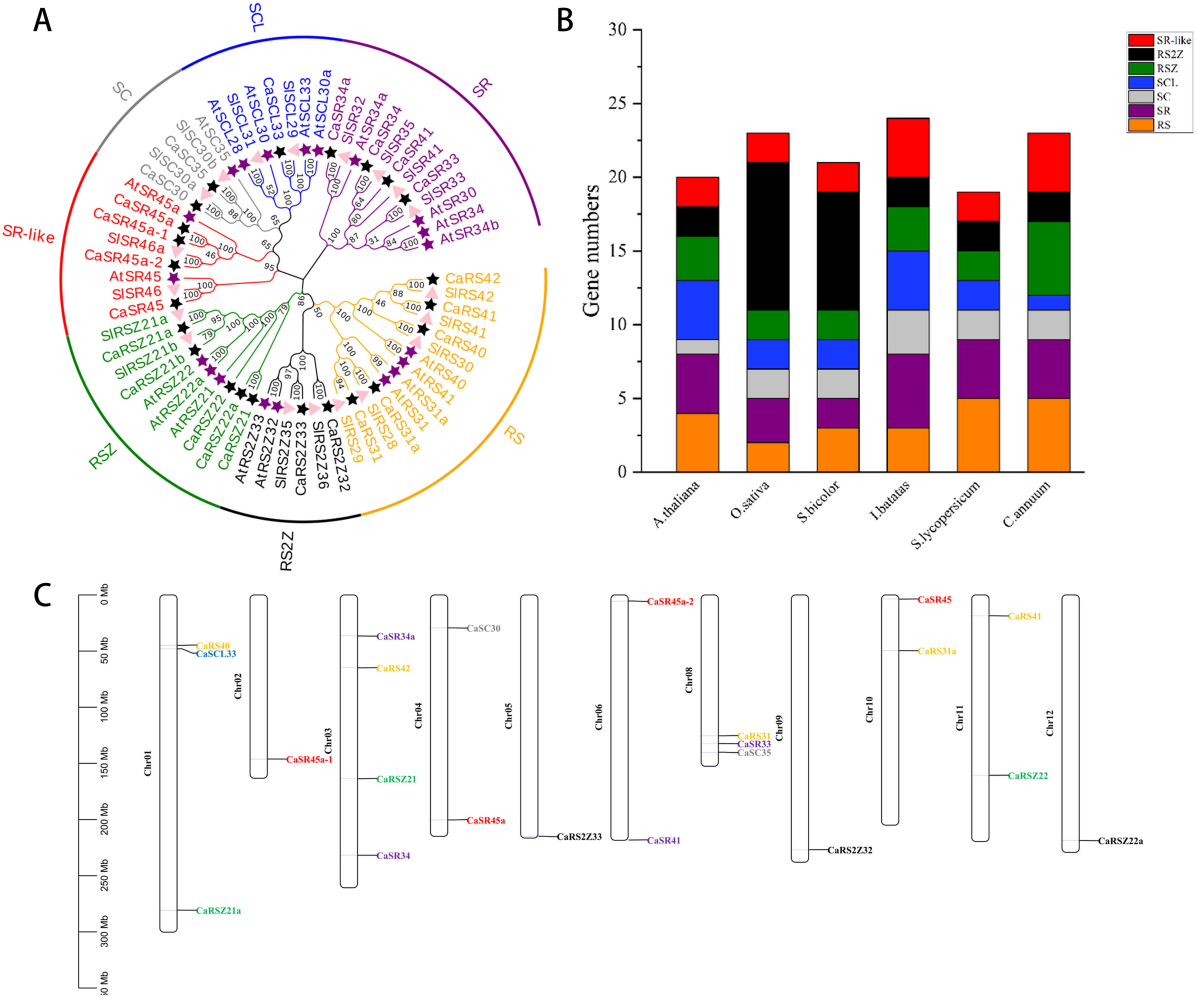
Phylogenetic tree and chromosome distribution of SR genes in hot pepper. **(A)**, Phylogenetic tree of SR proteins in rice, tomato, and hot pepper. RS, RSZ, SR, SCL, SC, SR-like, and RS2Z represent the seven distinct subfamilies. The purple star represents *Arabidopsis*, the pink triangle represents tomato, and the black star represents hot pepper. **(B)**, Number of SR genes in hot pepper, *Arabidopsis*, rice (*Oryza sativa*), sorghum (*Sorghum bicolor*), sweet potato (*Ipomoea batatas*), and tomato. **(C)**, Distribution of SR gene in hot pepper on chromosome; different colors indicate different subfamilies.

In order to explore the evolutionary and phylogenetic relationships among hot pepper, *Arabidopsis*, and tomato, MEGA11.0 was applied to construct a phylogenetic tree by comparing the full-length sequences of 62 SR proteins of the three species (**Figure 1A**). A systematic nomenclature of SR genes in hot pepper was made based on evolutionary relationships with AtSRs and SlSRs, and the 23 SR proteins of hot pepper are classified into seven known subfamilies, namely, SR, SC, SCL, RSZ, RS, RS2Z, and SR-like. SR and SR-like subfamilies contain four proteins each, RS and RSZ subfamilies contain five proteins each, and RS2Z and SC subfamilies contain two proteins each, while the SCL subfamily contains only one protein.

It is noteworthy that hot pepper has a large RS subfamily with more members, compared with other species listed here, and only one gene encoding the SCL protein in this genome (**Figure 1B**). This result is similar to the RS and SCL subfamilies of tomato SR proteins, suggesting a relative conserved in SR protein evolution in the same genus (Rosenkranz et al., 2021). In hot pepper, 22 SR genes were distributed on 11 of the 12 chromosomes, no SR gene on chromosome 7 (Chr07). Furthermore, the chromosomal location of CaRSZ21b could not be specified currently (**Figure 1C**). Among the 11 chromosomes, Chr04 contains the most SR genes, four, followed by Chr01 and Chr08 each carrying three SR genes. Chr04, Chr06, Chr10, and Chr11 each have two SR genes, while Chr02, Chr05, Chr09, and Chr12 each contain only one SR gene.

### 2.2 Conserved motif and gene structure analysis

**Figure 2:**
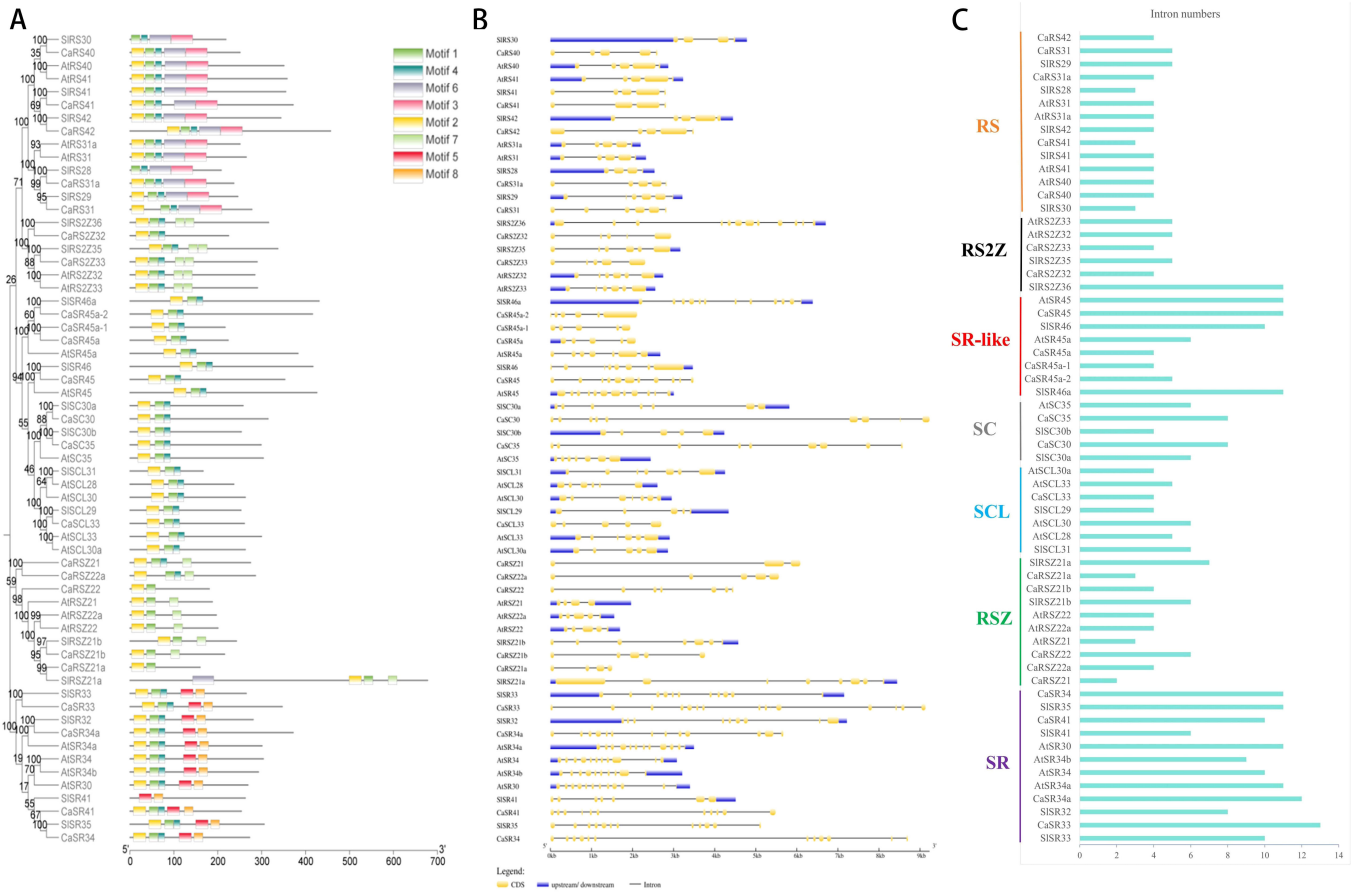
Analysis of gene structure and conserved motifs of SR proteins in hot pepper, *Arabidopsis*, and tomato. **(A)**, Conserved motifs of SR proteins. **(B)**, Gene structures of SR genes. **(C),** Intron numbers of SR genes.

The server MEME was used to analyze conserved motifs of SR proteins in three species and allowed differentiation up to eight amino acid motifs. As shown in **Figure 2A**, SR proteins of all three species contain two to five motifs. Moreover, SR proteins belonging to the same subfamily contain almost the same motifs. A total of 59 SR proteins contained Motif 1 and Motif 2, except RS30, RS28, and SR41 of tomato. Interestingly, motif 3 exists only in the RS subfamily, while motif 5 and motif 8 exist only in the SR subfamily.

The exon/intron arrangement of the coding sequence in the SR gene genome sequence of hot pepper, *Arabidopsis*, and tomato was further analyzed using the online tool GSDS (**Figure 2B**). The length of the coding region of the CaSR gene ranges from 1.5 kb to 9.3 kb, consistent with the length of tomato SR genes, ranging from 2.5 kb to 8.5 kb, which is generally longer than that of *Arabidopsis* (lengths of *Arabidopsis* SR genes range from 1.5 kb to 4 kb). The number of introns in the SR gene of hot pepper ranged from 2 to 14 (**Figure 2C**). The intron number in each gene of the RS subfamily did not change obviously. There are three to five introns in each gene of the RS subfamily in hot pepper. Also, there are the same numbers of intron in the tomato RS subfamily. Moreover, each gene in the *Arabidopsis* RS subfamily contained four introns. Each gene of the RS2Z subfamily in hot pepper has four introns, and this number is five in the *Arabidopsis* RS2Z subfamily, while that of the tomato RS2Z subfamily ranges from 5 to 11. The intron numbers change significantly in the SR-like subfamilies in all three species, ranging from 4 to 10 introns in hot pepper, 6 to 11 in *Arabidopsis*, and 7 to 11 in tomato, respectively. SC subfamily genes in hot pepper contained more introns (7 to 8) than their homologous genes in *Arabidopsis* and tomato, which are 6 and ranging from 4 to 6 each, respectively. The single member, CaSCL33, in hot pepper has 4 introns, and its homologous genes in *Arabidopsis* and tomato have 4 to 5 and 4 to 6 introns, respectively. Gene members of the RSZ subfamily in hot pepper have 2 to 6 introns each, and those of *Arabidopsis* and tomato have 3 to 4 and 6 to 7, respectively. Generally, the SR subfamily tends to contain more introns than other subfamilies; the intron numbers of the SR subfamily members range from 10 to 14 in hot pepper, 9 to 11 in *Arabidopsis*, and 6 to 11 in tomato.

In summary, SR proteins attributed to the same subfamily contain the same conserved motifs, suggesting that the functions of these proteins are relatively conversed in the plant kingdom during the evolutionary process (Chen et al., 2024). Thus, the gene structures of SR genes varied more obviously when compared with their resulting amino acid sequences they produced, mainly because of introns with different features they embrace.

### 2.3 Cis-acting elements analysis of the SR genes in hot pepper

**Figure 3:**
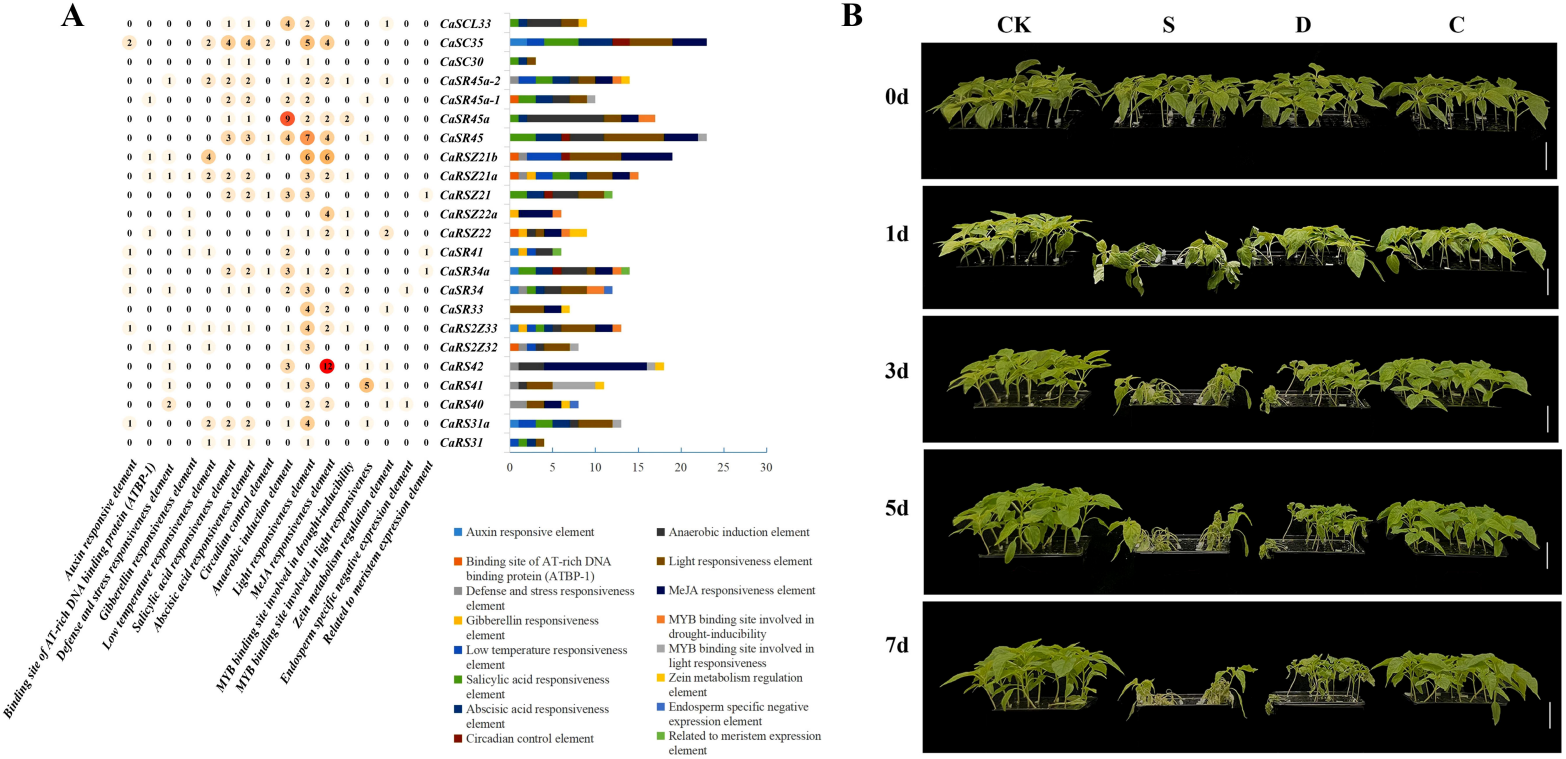
Cis-acting elements of CaSR genes and stress response of hot pepper seedlings. **(A)**, Numbers of cis-elements in CaSR gene promoters. **(B),** Seedlings growing under normal conditions and those treated with salt (S), drought (D), and cold (C) for 1 day (1d), 3d, 5d, and 7d.

Gene expression is importantly linked to cis-acting elements in its promoter (Oudelaar and Higgs, 2021). Therefore, PlantCARE software was used to predict cis-acting regulatory elements at the 1,500-bp promoter upstream of 23 SR genes in hot pepper (**Figure 3A**). The analyses showed that two well-known housekeeping cis-acting elements, CAAT-box and TATA-box, were detected in all SR genes, except for the CaRS42 gene, in which no TATA-box housekeeping cis-acting regulatory element was detected (**Supplementary Table 4**). Most of the CaSR genes contained light-responsive elements (20/23, 86.96%) and anaerobic-responsive elements (15/23, 65.22%), which are essential for light and anaerobic induction, respectively. A considerable number of CaSR genes contain stress-related elements, such as defense and stress response elements (8/23, 34.78%), low-temperature response elements (9/23, 39.13%), and MYB binding site involved in drought induction (8/23, 34.78%).

Interestingly, a large number of hormone response elements were found in most CaSR genes, such as salicylic acid response elements (14/23, 60.87%), abscisic acid response elements (14/23, 60.87%), MeJA response elements (13/23, 56.52%), and growth hormone response elements (5/23, 21.73%). Furthermore, some of CaSR genes contain response elements related to growth and development such as endosperm expression, meristem expression, zein metabolism regulation, and circadian control. Taken together, there is enrichment of cis elements involved in plant growth, development, and stress response in CaSR genes, indicating that they may play important roles in these processes.

### 2.4 Tolerance of hot pepper seedlings under abiotic stresses

Lots of cis-acting regulatory elements related to abiotic stress responses, such as drought and low temperature, were detected in the SR genes of hot pepper. To further explore these responses, we subjected hot pepper seedlings at the four-true-leaf stage to various abiotic stress conditions, including salt, drought, and cold. As shown in **Figure 3B**, stress treatments, especially salt and drought, resulted in noticeable inhibition and damage to the seedlings. After 8 days of continuous salt stress, most seedlings had died, while plants exposed to drought stress gradually withered and died. Cold-treated plants also exhibited inhibited growth, with plant height notably lower than that of the control group.

### 2.5 Expression profile of SR genes in hot pepper

**Figure 4:**
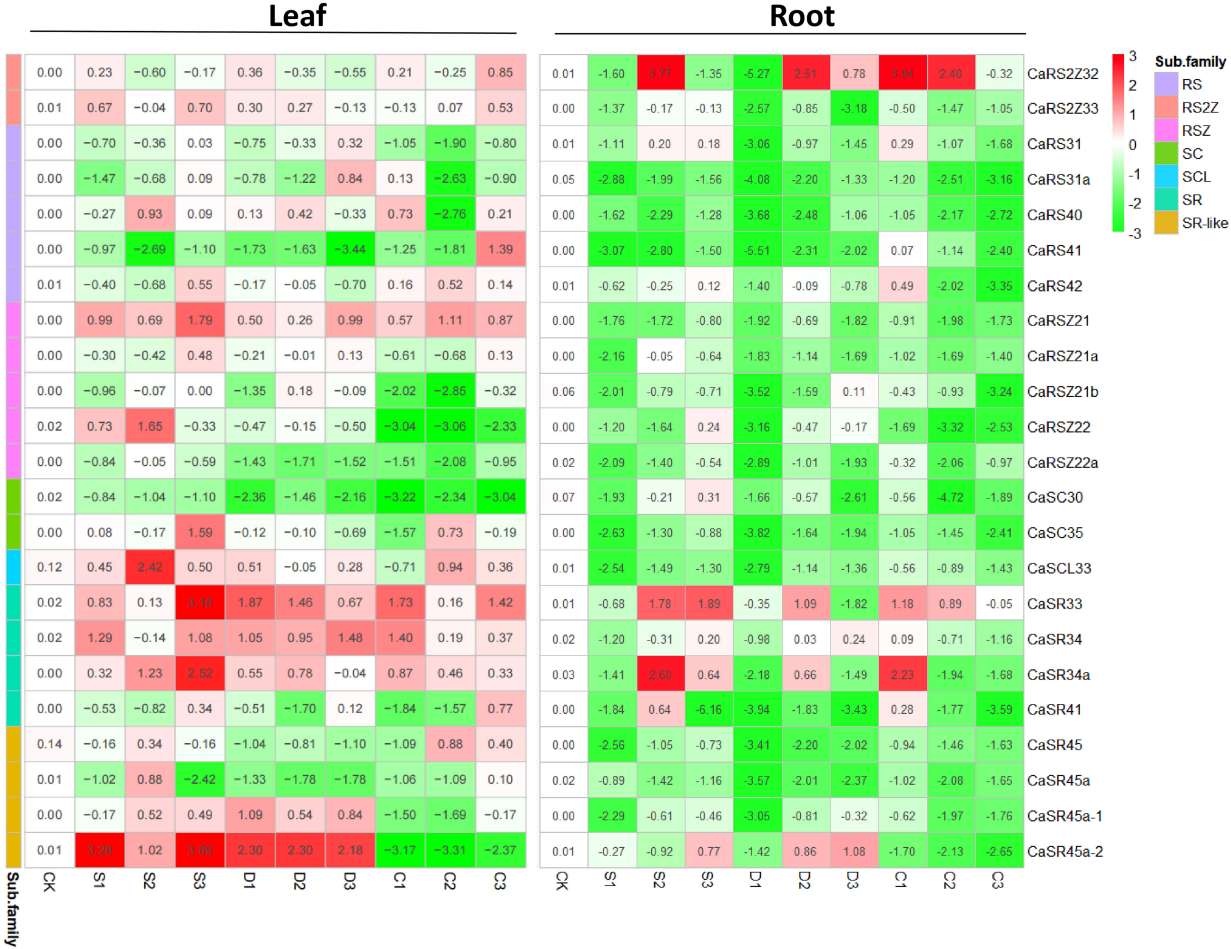
Expression of CaSR genes. Relative expression levels of CaSR genes are presented in a 2^(x) style, with the basic expression level of each gene under control conditions set to 1, (i.e., 2^0). The number and the corresponding color in each cell in the heatmap indicate the intensity of induction (red) or suppression (green) of gene expression, compared with its expression level under normal conditions. Information of cDNA samples is indicated at the top and bottom of the gel panels. CK, control; S, salt stress treatment; D, drought stress treatment; C, cold stress treatment. Also, the numbers 1, 2, and 3 denote different treatment times, 1 day, 2 days, and 3 days.

Since SR proteins are known to play critical roles in plant growth and stress resistance, we examined how these proteins respond to environmental stress at the gene transcription level (Xing et al., 2015; Albaqami et al., 2019). In leaves, *CaRSZ21*, *CaSR33*, *CaSR34*, and *CaSR34a* were generally upregulated in response to various abiotic stress treatments (**Figure 4**). In contrast, *CaSRZ22a* and *CaSC35* tended to be repressed by all stress types. Notably, *CaSR45-2* exhibited a differential response to osmotic versus cold stress: it was highly induced by salt and drought stress but significantly repressed under cold conditions.

Additionally, the expression levels of several SR genes appeared to vary with the duration of the stress treatment. For instance, CaRS41 was induced in leaves after 3 days of cold stress but generally decreased under other conditions and after 1 or 2 days of cold exposure. Another gene from the same subfamily, CaRS40, was significantly repressed only after 2 days of cold stress (C2), with slight upregulation or downregulation observed in most other treatments. This suggests that transcriptional regulation of SR genes is both stress-type and duration-dependent.

Interestingly, the expression patterns of SR genes in the root tissue of hot pepper seedlings differed markedly from those observed in leaves. Most SR genes in the roots were generally downregulated across various stress types and durations. However, a few genes, such as *CaRS2Z32*, *CaSR33*, *CaSR34a*, and *CaSR45a-2*, showed sharp upregulation at specific time points following abiotic stress treatments. This indicates that the root-specific response of SR genes to environmental stress may involve unique regulatory mechanisms compared with the leaf tissue.

### 2.6 Splicing of SR genes in different tissues of hot pepper under normal and stress conditions

**Figure 5:**
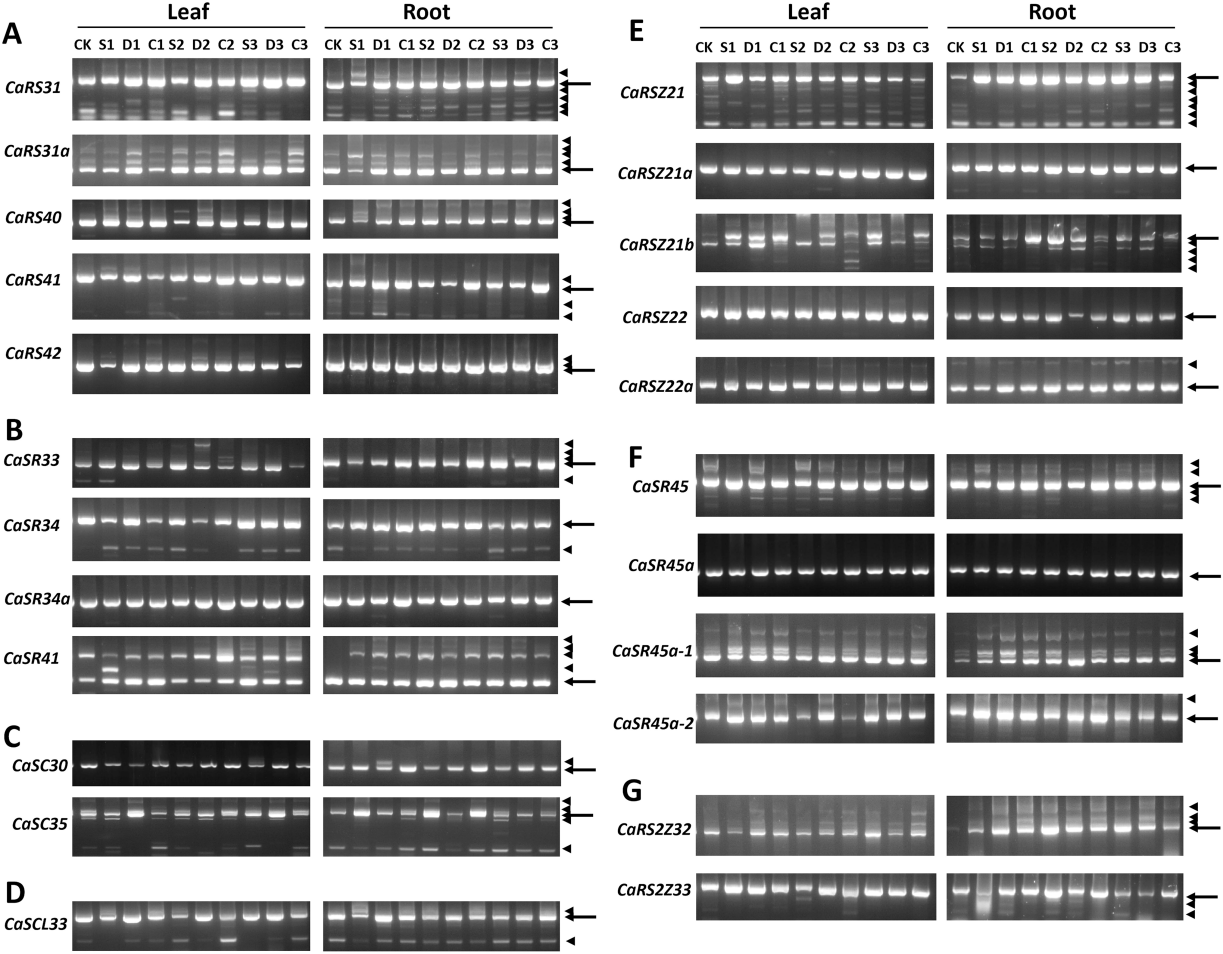
Splicing patterns of 23 SR genes in hot pepper in different environments. **(A)**, RS subfamily. **(B)**, SR subfamily. **(C)**, SC subfamily. **(D)**, SCL subfamily. **(E)**, RSZ subfamily. **(F)**, SR-like subfamily. **(G)**, RS2Z subfamily. Information of cDNA samples is indicated in uppermost part of the gel panels. CK, control; S, salt stress treatment; D, drought stress treatment; C, cold stress treatment. Also, the numbers1, 2, and 3 denote different treatment times, 1 day, 2 days, and 3 days, respectively. The arrow indicates a constitutively spliced isoform, while the arrowhead indicates an alternatively spliced isoform.

Dynamic regulation of AS in coding genes of SR proteins has been frequently observed across the plant kingdom (Palusa et al., 2007; Chen et al., 2022, 2024). In this study, we comprehensively analyzed the splicing patterns of all SR genes in both leaf and root tissues using RT-PCR. There were 19 out of the 23 SR genes that exhibited dynamic AS regulations under different environmental conditions, while the remaining four genes, namely, *CaSR34a*, *CaRSZ21a*, *CaRSZ22*, and *CaSR45a*, were prone to be constitutively spliced only, and no AS event was detected under the conditions used here (**Figure 5**).

In general, the SR genes that underwent AS exhibited varying splicing patterns between different tissues and under different environmental conditions. The relative abundance of certain alternatively spliced mRNA isoforms fluctuated with environmental changes. For example, *CaRS31*, *CaSR34*, *CaRSZ21b*, and *CaSR45a-1* exhibited changes in the intensity of their spliced isoforms based on environmental stress. This suggests that AS increases the complexity of the transcriptome, with some genes producing more transcripts under stress conditions. For instance, AS was observed in all RS subfamily genes in leaves. *CaRS31*, *CaRS31a*, and *CaRS40* produced six, three, and three transcripts, respectively, while *CaRS42* underwent AS only in leaves, not in roots. Additionally, the splicing pattern of *CaSR41* in the SR subfamily appeared to be highly sensitive to stress treatment in both leaf and root tissues. *CaSC35*, a member of the SC subfamily, generated five different transcripts, with the relative abundance of the constitutively spliced isoform fluctuating under different environmental conditions. The sole member of the SCL subfamily, *CaSCL33*, produced four AS transcripts.

In the CaRSZ subfamily, *CaRSZ21* generated multiple AS mRNA isoforms, with *CaRSZ21b* producing fewer transcripts than *CaRSZ21*, and the relative abundance of these transcripts varied significantly under different environmental conditions. Furthermore, three out of the four SR-like subfamily members underwent AS, while both members of the RS2Z subfamily were alternatively spliced.

In conclusion, most CaSR genes exhibited AS under both normal and abiotic stress conditions, with varying intensities of AS regulation observed between leaf and root tissues, as well as under different environmental conditions. This dynamic regulation of AS suggests that SR proteins play a crucial role in responding to environmental stress in hot pepper.

### 2.7 Sequence and splicing site analysis of some CaSR genes splicing variants

To investigate the sequence composition and AS events of specific splicing variants, we isolated and amplified PCR fragments from four SR genes (*CaRS31a*, *CaRS40*, *CaRSZ22a*, and *CaSR45a-1*). These fragments were cloned into pESI-blunt simple vectors for sequencing analysis. By comparing the sequence variants to the constitutive splicing isoforms, we identified various AS patterns, including intron retention, alternative 5′ splice site (5′SS) events, and alternative 3′ splice site (3′SS) events. For instance, intron retention was observed in the second intron of *CaRS31a*, the third intron of *CaRSZ22a*, and the first and fourth introns of *CaSR45a-1*. We detected 5′SS events in the first intron of *CaRSZ22a* as well as in the first and third introns of *CaSR45a-1*. Selective exon splicing also occurred in *CaRS31a*, *CaRS40*, and *CaSR45a-1*. Notably, we identified two separate AS events happened together in a single intron of *CaSR45a-1*, which involved alterations at both the 5′ and 3′ splice sites of the third intron (**Supplementary Figure 1**, **Supplementary Table 5**).

Based on sequencing results, we observed that pre-mRNA splicing in CaSR genes utilizes both typical U2-type splice sites (GT-AG) and atypical splice sites. For instance, in splice isoform 2 of *CaRSZ22a*, a larger intron is produced due to a typical U2-type splice site (GT-AG), while in splice isoform 1 of *CaSR45a-1*, a larger exon is formed due to a similar GT-AG splice site. Notably, multiple atypical splice sites were identified across all four splice variants (**Supplementary Table 6**). The use of these atypical splice sites contributed to the formation of additional exons or introns, further increasing the diversity of the splice variants.

### 2.8 Comparison of AS events of homologous SR proteins from different plant species

Although previous studies have shown that pre-mRNAs of SR proteins are highly susceptible to AS (Duque, 2011), to our knowledge, no research has yet compared whether AS occurs at similar regions of pre-mRNAs across different plant species. To address this, we mapped the locations of AS events in homologous SR genes from various plants, enabling a comparative analysis of these events across species.

**Figure 6:**
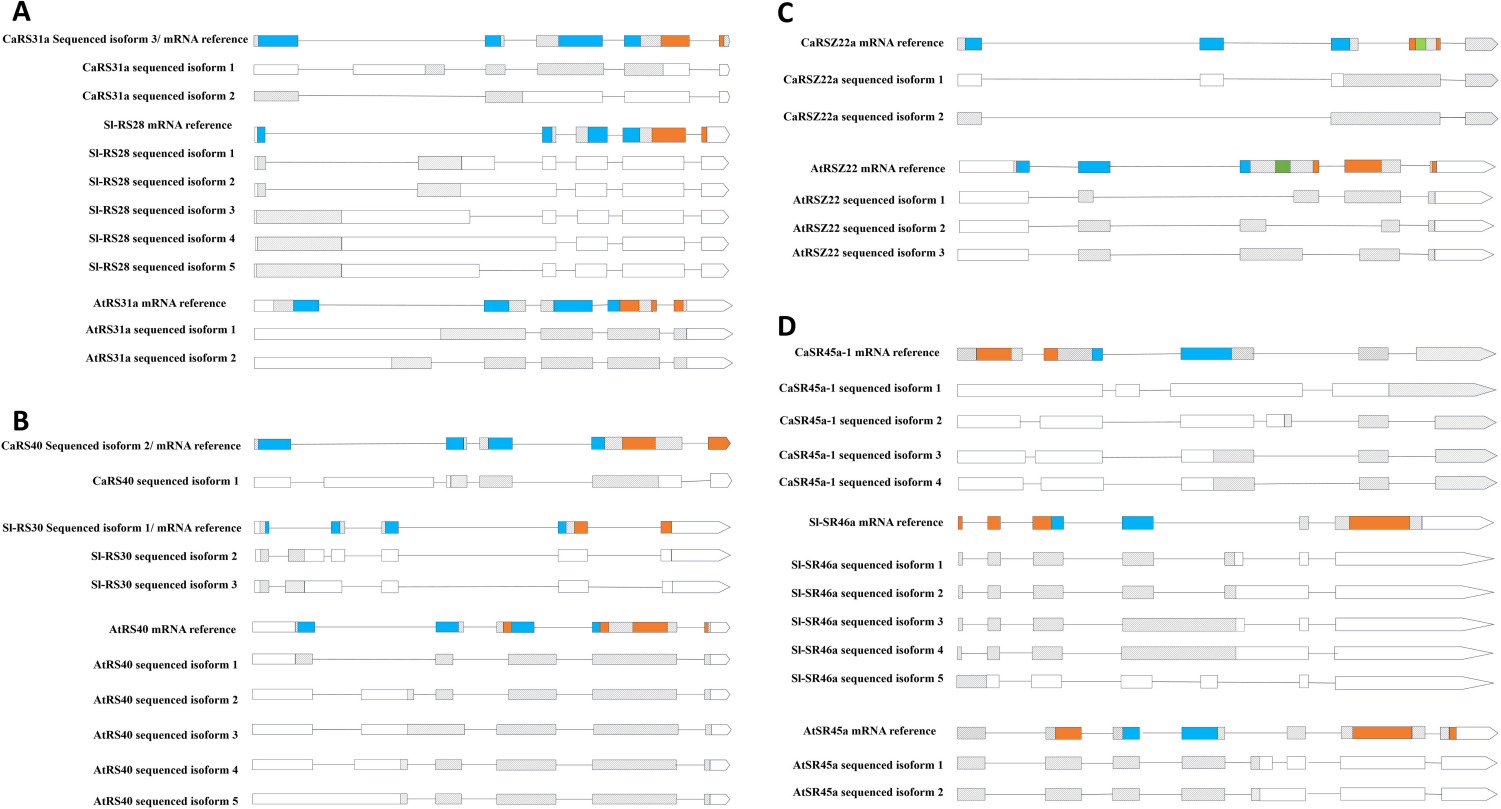
Comparison of AS patterns of SR homologous genes in three different plant species. **(A)**, RS31a from hot pepper, tomato, and *Arabidopsis*. **(B)**, RS40 from hot pepper and *Arabidopsis* and RS30 from tomato. **(C)**, RSZ22 from hot pepper and RSZ22 from *Arabidopsis*. **(D)**, RS45a-1 and SR46a and SR45a from hot pepper, tomato, and *Arabidopsis*. The gray box stands for exon on the CDS (coding sequence) region, and the white box stands for exon on the UTR (untranslated region). The line represents intron. The relative position of the encoding sequence for specific functional domains, such as RRM, RS/SR, and ZnK, is marked with different colors on the reference mRNA.

As shown in **Figure 6**, *CaRS31a* undergoes AS at the first and third introns, regions that are part of the coding sequence for the RRM domain in the SR protein (assuming constitutive splicing and translation). AS in this region can lead to changes in the coding sequence, potentially resulting in an altered amino acid sequence. Similarly, the pre-mRNAs of its homologous proteins in *Arabidopsis* and tomato also show a tendency for AS within the RRM-coding region. For *CaRS40*, AS events occur in mRNA regions encoding both the RRM and RS/SR domains, while in its homologs in *Arabidopsis* and tomato, AS is restricted to the RRM-coding region. In the case of *CaRSZ22a*, AS events occur in the third intron, which links the RRM and RS/SR domain-coding sequences, potentially altering the start of the coding sequence. In *Arabidopsis*, the AS window in *AtRSZ22* spans a broader range within this region. Interestingly, AS events in *CaSR45a-1* are dispersed across different introns, a pattern also observed in its tomato homolog, *SlSR46a*, whereas *AtSR45a* in *Arabidopsis* exhibits AS in narrower regions flanked by the coding sequences for the RRM and RS/SR domains.

**Figure 7:**
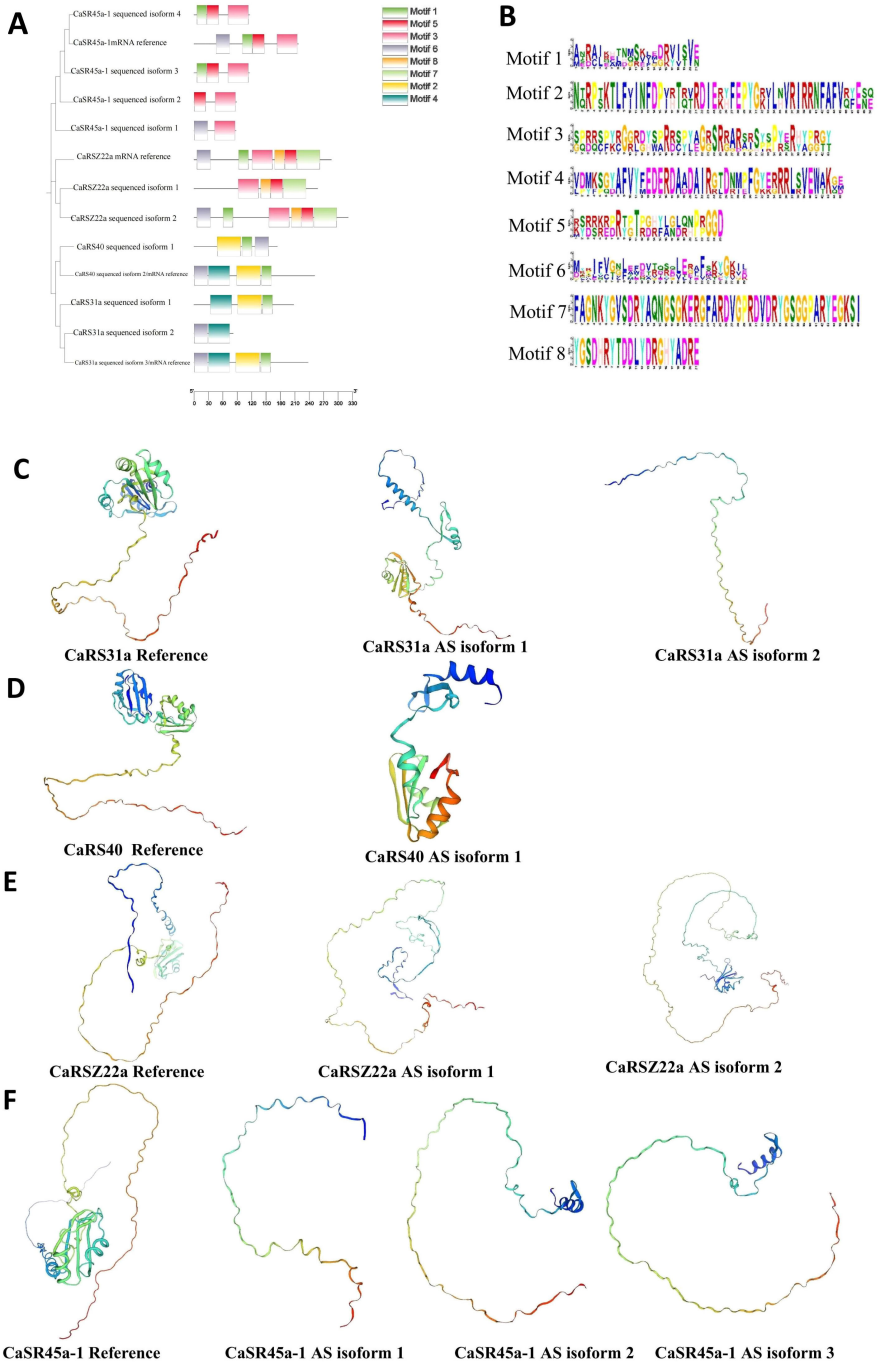
Component and 3D structure changes of potential CaSR protein variants generated form alternatively spliced mRNA isoforms. **(A)**, Conserved motifs of different CaSR protein variants. **(B)**, Amino sequence of motifs 1–8. **(C)**, 3D structure of CaSR31a protein variants. **(D)**, 3D structure of CaSR40 protein variants. **(E)**, 3D structure of CaRSZ22a protein variants. **(F)**, 3D structure of CaSR45a-1 protein variants.

We analyzed the potential amino acid (AA) products generated from the alternatively spliced mRNA isoforms and found that these variants may lack some conserved motifs compared with the constitutively spliced isoform. For instance, the constitutively spliced mRNA of *CaSR45a-1* encodes an AA sequence containing conserved motifs 1, 3, 5, and 6. In contrast, potential isoform 1 lacks motifs 1 and 5, containing only motifs 3 and 6; isoform 2 contains motifs 3 and 5; and isoforms 3 and 4 contain motifs 1, 3, and 5. Further 3D structure analysis reveals that the potential proteins from alternatively spliced isoforms of CaSR genes have truncated AA chains, resulting in shorter protein structures compared with those translated from the constitutively spliced mRNA isoforms (**Figure 7**). This highlights the impact of AS on the structural and functional diversity of SR proteins across plant species.

## 3 Discussion

AS of pre-mRNA, a critical posttranscriptional regulatory mechanism in eukaryotes, plays significant roles in plant growth, development, and stress adaptation (Reddy and Shad Ali, 2011; Black, 2003). Serine/arginine (SR)-rich proteins are an important class of splicing factors that play a key role in the execution and regulation of pre-mRNA splicing by binding to the splicing enhancer of pre-mRNAs to regulate splicing site selection (Zahler et al., 1992; Duque, 2011). Initial studies of plant SR proteins in *Arabidopsis* (Reddy, 2004; Lorković and Barta, 2002) laid the foundation for subsequent research across other plant species, greatly expanding our understanding of SR protein functions and mechanisms (Gu et al., 2020; Chen et al., 2020b, 2024, 2022).

Here, we identified 23 SR protein-encoding genes from the hot pepper genome. These SR proteins are unevenly distributed across the 12 chromosomes of hot pepper, similar to SR protein distributions observed in other plants. As with many plant species, CaSR proteins can be classified into seven subfamilies: SR, RSZ, SCL, RS, RS2Z, SC, and SR-like. Interestingly, our phylogenetic analysis revealed a high degree of homology between CaSR proteins and those of *Arabidopsis* and tomato. Notably, the RS subfamily in hot pepper contains more members, whereas the SCL subfamily is represented by only one gene, CaSCL33. This discrepancy might reflect species-specific RNA regulatory needs, as certain RS genes show an elevated expression during early fruit development (Sun and Xiao, 2015; Rosenkranz et al., 2021).

Gene structure analysis across *Arabidopsis*, tomato, and hot pepper revealed that the intron number of most members of the same SR subfamily changed in a relatively narrow range. Furthermore, we observed diverse regulatory elements within the 1,500-bp upstream promoter regions of CaSR genes, including light-responsive elements, abiotic stress-response elements, hormone signaling, and growth-related elements, which could influence the expression patterns of these genes (Oudelaar and Higgs, 2021).

Our results showed that SR gene expression in hot pepper responds dynamically to various abiotic stresses, including continuous salt, drought, and periodic cold stress. In leaves, most SR subfamily genes were upregulated under these stresses, except CaSR41, which was downregulated. This suggests a potential role of SR proteins in stress resilience. Specifically, the expression of *CaSR33*, *CaSR34*, and *CaSR34a* was consistently induced across different environmental stresses, highlighting their possible functions in stress resistance, aligning with findings from other plants such as *Arabidopsis*, sweet potato, and pine, where homologous SR genes are also stress-induced (Tanabe et al., 2007; Chen et al., 2022, 2024). Functional studies further support this, as overexpression of the cassava SR subfamily gene MeSR34 in *Arabidopsis* significantly enhanced salt tolerance in transgenic lines (Gu et al., 2020).

SR genes are known not only to regulate the splicing of other genes but also to undergo AS themselves, modulating plant development and stress responses (Xie et al., 2022; Oudelaar and Higgs, 2021). In our analysis, 19 out of 23 SR genes in hot pepper exhibited AS, generating approximately three times as many transcripts, thereby increasing transcriptomic complexity. We found a considerable number of intron retention events in some CaSR genes, consistent with conclusion of previous studies that intron retention emerged as the most prevalent AS type among CaSR genes, which is the most prevalent AS type in the plant kingdom (Wang and Brendel, 2006).

The AS patterns of SR genes also varied across different tissues. For example, most splice variants of *CaRS2Z32* showed a higher expression in roots than in leaves. Additionally, *CaSR45a-1* expressed four splice variants, whereas *CaSR45a-2* produced two, with varying mRNA abundances depending on tissue type and environmental conditions. Interestingly, RS subfamily genes were particularly prone to AS, except for *CaRS42*, which did not undergo AS in roots. Similar trends were observed in sweet potato and pine, where RS genes displayed high AS activity (Chen et al., 2024, 2022). In contrast, certain RSZ subfamily members, such as *CaRSZ21a* and *CaRSZ22*, did not exhibit significant AS in either leaf or root tissues under different conditions, echoing findings in *Arabidopsis*, cassava, sweet potato, and pine (Chen et al., 2022, 2024; Palusa et al., 2007; Gu et al., 2020).

Our results indicate that the AS patterns of SR genes are, to a certain extent, conserved across plant species. It has been demonstrated that SR proteins in plants are functionally important and conserved (Palusa et al., 2007). At the same time, most of SR proteins underwent AS regulation frequently (Chen et al., 2022, 2024; Palusa et al., 2007; Gu et al., 2020). Here, we demonstrated that AS events on SR genes appear to be largely conserved across different plants, despite significant genomic sequence divergence among homologous genes. For example, comparative AS analyses for several CaSR genes and their homologous genes in *Arabidopsis* and tomato (another Solanaceae species) suggest that many AS events occur at pre-mRNA sequences that would translate into the RRM domain if constitutively spliced. This finding further supports the idea of a conserved AS regulatory mechanism among different plants, pointing to evolutionary stability in SR protein functions and regulatory mechanism.

## 4 Conclusion

A total of 23 SR genes were identified and characterized from the hot pepper genome (zunla-1). They were categorized into seven subfamilies. The gene structures and conserved motifs of SR genes from different species but belonging to the same subfamily were largely conserved. Among the CaSR genes, we identified a number of the cis-acting elements associated with growth, development, and stress response, which may play important roles in these processes. Subsequently, we found that most of the SR genes in leaves are induced and upregulated at some point in time, whereas most of the SR genes in roots showed a downregulated expression pattern. Most of CaSR genes undergo AS under both normal and abiotic stress conditions. We identified multiple AS events and novel splice sites in the sequences of CaSR gene splice variants. Furthermore, by cross-species comparison, we found that AS regulations of SR protein were carried out at the region where it would be translated into the RRM domain in the resulting protein.

## 5 Materials and methods

### 5.1 Plant material and stress treatments

We sowed hot pepper seeds in seedling trays containing a mixture of nutrient soil and vermiculite (2:1). After the seedlings emerged, we gently washed the nutrient soil from the roots with water and transplanted the seedlings into 1/2 Hoagland nutrient solution. The seedlings were then incubated for 2 weeks at 25°C in a growth chamber with a 12-h light/dark cycle to prepare them for abiotic stress treatments.

Drought and salt stresses were induced by adding 20% PEG6000 and 200 mM NaCl, respectively. Leaves and roots were collected at 1, 2, and 3 days after the start of each treatment. For cold stress, we simulated a low-temperature environment with a temperature cycle of 25°C from 9:00 to 21:00 and 10°C from 21:01 to 8:59, and samples were collected at 1, 2, and 3 days following treatment initiation. For each time point under normal, drought, salt, and cold conditions, three biological replicates were collected. The harvested leaves and roots were immediately snap-frozen in liquid nitrogen and stored at −80°C for RNA extraction.

### 5.2 Genome-wide identification of SR genes in hot pepper

To investigate the SR gene family in hot pepper, we used the genome-wide data (v2.0) of “Zunla-1,” downloaded from the hot pepper Genome Database (peppersequence.genomics.cn) as our reference genome (Qin et al., 2014). *Arabidopsis* SR sequences were obtained from the Arabidopsis database (TAIR10, arabidopsis.org), while SR sequences for tomato were sourced from the Phytozome13 database (phytozome.doe.gov).

Using the SR proteins from *Arabidopsis* and tomato as reference points, we identified target genes within the hot pepper genome using the BLAST function in TBtools software (Chen et al., 2020a). The structural integrity of the RRM1 and ZF_CCCH domains was confirmed via the InterPro Platform (ebi.ac.uk) (Paysan-Lafosse et al., 2023) and the SMART database (smart.embl-heidelberg.de) (Schultz et al., 2000). Amino acid number (AA), molecular weight (MW), total average hydrophilicity (GRAVY), and isoelectric point (pI) were calculated using the Expasy website (web.expasy.org). Additionally, subcellular localization predictions were conducted using the online tool Cell-PLoc 2.0 (sjtu.edu.cn) (Chou and Shen, 2010).

### 5.3 Phylogenetic trees and chromosome localization

The phylogenetic analysis included SR proteins from three species: *Arabidopsis* (20 AtSRs), tomato (19 SlSRs), and hot pepper (23 CaSRs). Protein sequences were aligned using Clustal W, and the neighbor-joining (NJ) method with 1,000 bootstrap replications of MEGA11 was used to construct the phylogenetic tree (Kumar et al., 2018). For visualization, the evolutionary tree in Newick format was uploaded to Evolview (evolgenius.info). Based on the tree, systematic naming of CaSRs in hot pepper was assigned according to their evolutionary relationships with AtSRs and SlSRs. Chromosomal distribution of CaSRs was then visualized using TBtools software.

### 5.4 Conserved motif and gene structure analysis of the CaSR proteins

SR protein sequences from hot pepper, *Arabidopsis*, and tomato were analyzed for motif prediction using MEME Suite Version 5.4.1, with a maximum of eight motifs specified and default settings applied (Bailey et al., 2009). The results were then visualized using TBtools. For gene structure analysis, coding sequences (CDS) and genomic DNA sequences of SR genes from the three species were uploaded to GSDS (Gene Structure Display Server 2.0, gao-lab.org) to visualize intron–exon organization (Hu et al., 2015).

### 5.5 Analysis of cis-acting regulatory elements of the CaSR genes promoter

Annotation files of the hot pepper genome were imported into TBtools software to extract the 1,500-bp promoter regions upstream of each SR gene. These promoter sequences were then submitted to PlantCARE (PlantCARE database), a tool for identifying cis-acting regulatory elements in plant promoters, to predict the potential regulatory elements involved in stress responses and other biological processes (Lescot et al., 2002).

### 5.6 RNA isolation, RT-PCR analysis, and qRT-PCR analysis

Total RNA was extracted from the leaves and roots of hot pepper seedlings using an RNA extraction kit (Cat#9769, Takara, Japan) according to the manufacturer’s protocol. The extracted RNA was reverse-transcribed into complementary DNA (cDNA) using a DNA synthesis kit (R323, Vazyme, Nanjing, China). This cDNA served as the template for both reverse transcription polymerase chain reaction (RT-PCR) and quantitative RT-PCR (qRT-PCR). PCR primers were designed using SnapGene software (see **Supplementary Tables 2** and **3** for primer sequences).

The RT-PCR reaction was prepared in a 20-μL volume, which included 10 μL of 2× Hieff^®^ Robust PCR premix (Cat#101016ES03, YESEAN, Shanghai, China), 1 μL of forward primer (10 μM), 1 μL of reverse primer (10 μM), 1 μL of cDNA, and 7 μL of distilled water. The reaction program was set as follows: initial denaturation at 95°C for 3 minutes, followed by 40 cycles of denaturation at 95°C for 30 s, annealing at 56°C for 30 s, and extension at 72°C for 2 min. A final extension was performed at 72°C for 5 min. RT-PCR products were visualized on a 2% agarose gel using electrophoresis.

qRT-PCR was performed using SYBR Green Real-Time PCR Master Mix (Bio-Rad, USA) on a CFX96 Real-Time PCR system (Bio-Rad, USA). Each 10-μL reaction solution contained 5 μL of 2× Hieff^®^ qPCR SYBR^®^ Green Premix (Cat#11201ES08, YESEAN, Shanghai, China), 0.2 μL of forward primer (10 μM), 0.2 μL of reverse primer (10 μM), 0.5 μL of cDNA, and 4.1 μL of distilled water.

The expression levels were normalized to the internal reference gene CaUbi3 (Qin et al., 2018), and relative expression levels were calculated using the 2^−ΔΔCT^ method (Livak and Schmittgen, 2001).

### 5.7 Cloning and sequencing of splice variants

To detect specific gene sequences, primers were mixed with PrimeSTAR Max Premix (2×) (Cat#R045A, Takara, Japan), cDNA, and distilled water for RT-PCR. The RT-PCR procedure followed the same conditions as previously described. The PCR products were then purified using a gel extraction kit (Cat#CW2302M, Com Win Biotech, China) and subsequently cloned into the pESI-blunt simplex vector (Cat#10910ES20, Easen, China). The cloned products were sent to Sangon Biotech (China) for sequencing, and the sequencing results were analyzed using SnapGene software.

## Data Availability

The datasets presented in this study can be found in online repositories. The names of the repository/repositories and accession number(s) can be found in the article/**Supplementary Material**.
