# Supplementary figures and images for: Splicing and expression dynamics of SR genes in hot pepper (Capsicum annuum): regulatory diversity and conservation under stress
Source: Front Plant Sci. 2025 Jan 23;15:1524163. doi: 10.3389/fpls.2024.1524163 (PMC11798799; doi:10.3389/fpls.2024.1524163)

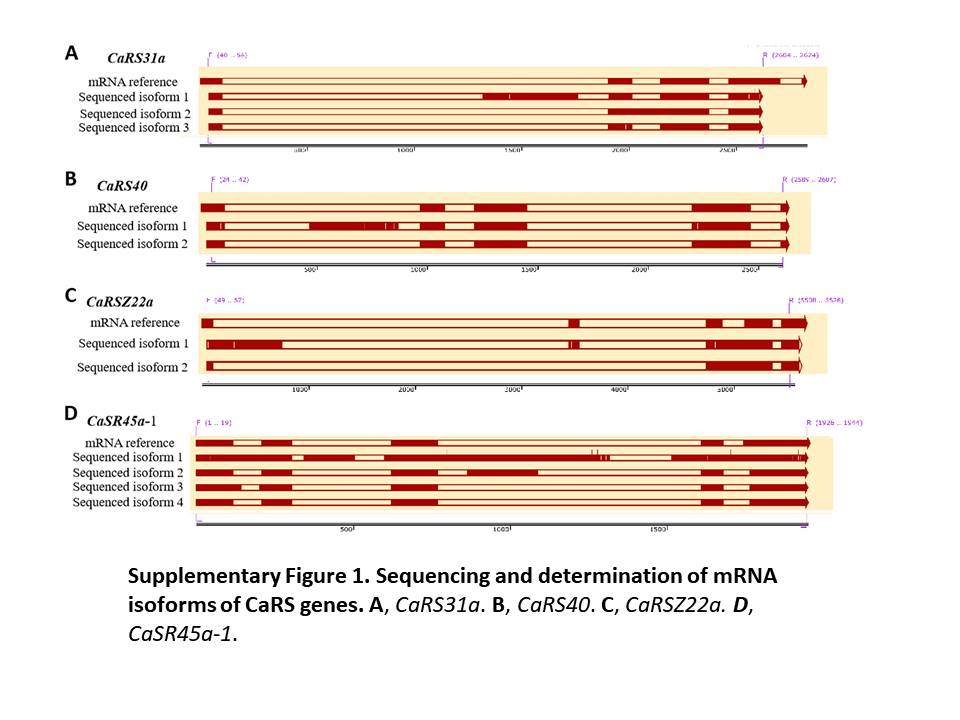

Supplement: Supplementary file 1 [file Image1.jpeg]
