# Supplementary material for: Splicing and expression dynamics of SR genes in hot pepper (Capsicum annuum): regulatory diversity and conservation under stress
Source: Front Plant Sci. 2025 Jan 23;15:1524163. doi: 10.3389/fpls.2024.1524163 (PMC11798799; doi:10.3389/fpls.2024.1524163)
Supplement: Supplementary file 2 [file Table1.docx]

| **Gene ID** | **Gene name** | **AA** | **MW/Da** | **PI** | **GRAVY** | **Subcellular localization** |
| --- | --- | --- | --- | --- | --- | --- |
| Capana08g001120 | CaRS31 | 278 | 32643.66 | 9.81 | -0.922 | Chloroplast. Nucleus. |
| Capana10g000784 | CaRS31a | 237 | 28386.74 | 10.08 | -1.221 | Nucleus. |
| Capana01g001361 | CaRS40 | 251 | 30065.61 | 10.69 | -1.338 | Nucleus. |
| Capana11g000560 | CaRS41 | 372 | null | null | -1.342 | Nucleus. |
| Capana03g002382 | CaRS42 | 457 | 51806.84 | 9.85 | -1.036 | Chloroplast. Nucleus. |
| Capana03g003623 | CaSR34 | 273 | 30730.09 | 11.01 | -1.059 | Nucleus. |
| Capana03g001832 | CaSR34a | 372 | 40909.91 | 8.92 | -0.529 | Nucleus. |
| Capana08g001537 | CaSR33 | 347 | 39892.36 | 8.25 | -0.326 | Nucleus. |
| Capana06g003060 | CaSR41 | 254 | 28646.58 | 9.93 | -0.628 | Chloroplast. |
| Capana09g002049 | CaRS2Z32 | 225 | 25739. | 9.11 | -1.56 | Cytoplasm. Nucleus. |
| Capana05g002416 | CaRS2Z33 | 290 | 32908.08 | 9.6 | -1.435 | Chloroplast. Nucleus. |
| Capana03g003059 | CaRSZ21 | 275 | 30878.83 | 8.37 | -1.11 | Chloroplast. Nucleus. |
| Capana12g002477 | CaRSZ22a | 286 | 32344.7 | 9.16 | -1.061 | Chloroplast. Nucleus. |
| Capana11g001347 | CaRSZ22 | 181 | 19550.53 | 5.93 | 0.16 | Chloroplast. Cytoplasm. |
| Capana00g003453 | CaRSZ21b | 216 | 24326.15 | 10.06 | 0.16 | Chloroplast. Nucleus. |
| Capana01g004055 | CaRSZ21a | 160 | null | null | -1.284 | Chloroplast. Nucleus. |
| Capana06g000376 | CaSR45a-2 | 416 | 50013.79 | 10.73 | -1.59 | Nucleus. |
| Capana02g002521 | CaSR45a-1 | 217 | 25288.07 | 11.38 | -1.465 | Chloroplast. Nucleus. |
| Capana04g002375 | CaSR45a | 224 | 26063.91 | 11.41 | -1.475 | Chloroplast. Nucleus. |
| Capana10g000216 | CaSR45 | 353 | 39737.56 | 12.26 | -1.305 | Chloroplast. Nucleus. |
| Capana04g001085 | CaSC30 | 315 | 36241.66 | 11.06 | -1.15 | Nucleus. |
| Capana08g002023 | CaSC35 | 299 | 34903.01 | 10.57 | -1.213 | Nucleus. |
| Capana01g001493 | CaSCL33 | 261 | 30393.51 | 11.4 | -1.603 | Chloroplast. Nucleus. |

**Supplementary table 1: Physicochemical properties of SR protein in capsicum.**
