# Supplementary material for: Splicing and expression dynamics of SR genes in hot pepper (Capsicum annuum): regulatory diversity and conservation under stress
Source: Front Plant Sci. 2025 Jan 23;15:1524163. doi: 10.3389/fpls.2024.1524163 (PMC11798799; doi:10.3389/fpls.2024.1524163)
Supplement: Supplementary file 3 [file Table2.docx]

| **Primer name** | **Forward primer (5’→3’)** | **Reverse primer (5’→3’)** |
| --- | --- | --- |
| *CaRS31* | GGAAGGATCCAGCGTGTTG | GCTCTTGACCTCCGAACAG |
| *CaRS31a* | CGCCAATCAGAGCTGGAAC | CAGGACTTGGTCGGTTCCTAC |
| *CaRS40* | TCTGGAGTTTGAAGCCCGC | TCAAGGTCGAGACCTCTCG |
| *CaRS41* | ATGGGAGAGTGGATAGGGTGG | GGAGCGAGATCTTTCACGAGC |
| *CaRS42* | TGCACGGCTGCTCTACTAC | GTCAGGACTTGGCCCATCT |
| *CaSR34* | AGTCGGTCGAGTAGGACAC | AGATGGCACTGGCGATCTG |
| *CaSR34a* | CGAGATGCAGAAGATGCC | ATTGGCTGCCTTCACCAC |
| *CaSR33* | TGGGTCGTCTAAGTCGGAC | TCCTGTTGGCATCACCTGG |
| *CaSR41* | AGTCGTTCAAGCAGGACG | TAAGCTTGGCAGATGGCG |
| *CaRS2Z32* | GGGACACTTGTCTTCACGG | GGTGATTCGCTCTCTCTACCC |
| *CaRS2Z33* | GCCGAGGTATGATGATCGG | CTCACTGCCTCTAGGAGAACC |
| *CaRSZ21* | GGTGGGTTATCTTGGGACG | AACGGTCGAAGGAAGGTGG |
| *CaRSZ22a* | TGGGATGTTACCGACCGTC | CGAGGGCGATCATATGGTC |
| *CaRSZ22* | GTCGAGGGTTTACGTAGGG | GACGATATGCTGGGGACCT |
| *CaRSZ21b* | ATCTGGACCCTAGGGTCAGTG | GGGTGTAGGCAGTCAAAGC |
| *CaRSZ21a* | CTTGATCCCAGGGTCACTG | CATCTCTGCCACGGTATGG |
| *CaSR45a-2* | CGTACTCCAGGAGGTCAAG | ACTGCGGCTCCTCTTAGAG |
| *CaSR45a-1* | TCTCGCTCTCCGTCACCTT | CTGTAACCGCGAGGGTAGT |
| *CaSR45a* | GGCAAGATCAAGGTCAAGGTC | ATACCTACCACGGTCACCAC |
| *CaSR45* | ACCGCCACCAGAGTCTAAG | TACGAGGAGGCGAACTGCT |
| *CaSC30* | TCGGTAGATCTGGTCCACC | CGACAATGGGGACAGAGT |
| *CaSC35* | TTCGGAAGAACTGGCCCTC | ATCGGGCACAGAGGTAGATCC |
| *CaSCL33* | TACAGCCCATCACCACCAAG | GGAGAACGATCTCCATCTGG |

**Supplementary table 2: RT-PCR primer sequence of SR gene in pepper**
