# Supplementary material for: Splicing and expression dynamics of SR genes in hot pepper (Capsicum annuum): regulatory diversity and conservation under stress
Source: Front Plant Sci. 2025 Jan 23;15:1524163. doi: 10.3389/fpls.2024.1524163 (PMC11798799; doi:10.3389/fpls.2024.1524163)
Supplement: Supplementary file 4 [file Table3.docx]

| **Primer name** | **Forward primer (5’→3’)** | **Reverse primer (5’→3’)** |
| --- | --- | --- |
| *CaRS31* | TGAGGGATGATGGCGAGAG | CATAGACTGGGCTACGAGG |
| *CaRS31a* | GAAGGATGACGATGAGAGGGG | CAGGACTTGGTCGGTTCCTAC |
| *CaRS40* | GGTCGTGACATGTCACCTG | CGGCCATACTCAGGACTTC |
| *CaRS41* | ATGACCGAGACCGATCTCG | TCATGCTTGGACTACGGCC |
| *CaRS42* | ATGGAAGAGACAGGCGGAG | TAGGAGCGCGATCTTTCCC |
| *CaSR34* | AGGAATATGACCGCAGTCG | CTTTTGCTCCTGCTACGAC |
| *CaSR34a* | TTGCTACGGTGGTGAAGGC | CCACCACCTGTCCCAACATTC |
| *CaSR33* | TGAACTTGCGCATGGTGG | TGAACTTGCGCATGGTGG |
| *CaSR41* | TGATTCTAGAGGCCGCAG | GACACAGAGCGTCCTTTC |
| *CaRS2Z32* | ATAGCCCAGCTCCTCGAAG | GGTGATTCGCTCTCTCTACCC |
| *CaRS2Z33* | TGACCCGAGAGACATGAGC | CTCACTGCCTCTAGGAGAACC |
| *CaRSZ21* | GCTGGAAAGAGACACAGGC | GGTTGGGCCTTGTTCACTG |
| *CaRSZ22a* | CCAGTGCTTCAAGTGTGGC | CCATAACGATCCCCACGTG |
| *CaRSZ22* | GAGTTACAACCCTCGTGG | AAGGTGACTCATAGCGCC |
| *CaRSZ21b* | TTCTAGAGGAGGAGGTGGTGGG | TGCGGTATCTAGGAGGGCT |
| *CaRSZ21a* | GTCCAAGCTATGGTCGCAG | CATCTCTGCCACGGTATGG |
| *CaSR45a-2* | GGTCCAGGAGAAGCTACTCAC | ACTGCGGCTCCTCTTAGAG |
| *CaSR45a-1* | GTGATCGTGGCAGGTATCGTG | CTGTAACCGCGAGGGTAGT |
| *CaSR45a* | CCGTGATGATTATGGGCGC | CTGCCACCACGAACATAG |
| *CaSR45* | AGGATCTCCTAGACGTGGG | GGTGTCCGATGACGTCTTG |
| *CaSC30* | GGATCCTCTGGTTGCTGT | CGACAATGGGGACAGAGT |
| *CaSC35* | AGCAGAAGCGTTGATCGC | AATGATGGGCTCCGTCTACG |
| *CaSCL33* | TGCACGTGACCATTCTCCTC | GGAGAACGATCTCCATCTGG |

**Supplementary table 3. qRT-PCR primer sequence of SR gene in pepper**
