# Supplementary material for: Splicing and expression dynamics of SR genes in hot pepper (Capsicum annuum): regulatory diversity and conservation under stress
Source: Front Plant Sci. 2025 Jan 23;15:1524163. doi: 10.3389/fpls.2024.1524163 (PMC11798799; doi:10.3389/fpls.2024.1524163)
Supplement: Supplementary file 6 [file Table5.docx]

| **Transcript name** | **Intron position** | **AS types** |
| --- | --- | --- |
| *CaRS31a* Isoform1 | 1^th^ | Alt. exon |
| *CaRS31a* Isoform2 | 2^th^ | IR |
| *CaRS40* Isoform1 | 1^th^ | Alt. exon |
| *CaRSZ22a* Isoform1 | 1^th^ | Alt. 3′-S |
| *CaRSZ22a* Isoform1 | 3^th^ | IR |
| *CaRSZ22a* Isoform2 | 1^th^ | ES |
| *CaRSZ22a* Isoform2 | 3^th^ | IR |
| *CaSR45a-1* Isoform1 | 1^th^ and 4^th^ | IR |
| *CaSR45a-1* Isoform1 | 2^th^ | Alt. exon and Alt. 5′-S |
| *CaSR45a-1* Isoform1 | 3^th^ | Alt. 5′-S and Alt. 3′-S |
| *CaSR45a-1* Isoform2 | 3^th^ | Alt. exon |
| *CaSR45a-1* Isoform2 | 4^th^ | ES |
| *CaSR45a-1* Isoform3 | 1^th^ | Alt. 3′-S |
| *CaSR45a-1* Isoform3 | 4^th^ | ES |
| *CaSR45a-1* Isoform4 | 4^th^ | ES |

**Supplementary table 5：Splicing types of CaRS31a, CaRS40, CaRSZ22a, and CaSR45a-1 alternative splicing variants.**
