# Supplementary material for: Splicing and expression dynamics of SR genes in hot pepper (Capsicum annuum): regulatory diversity and conservation under stress
Source: Front Plant Sci. 2025 Jan 23;15:1524163. doi: 10.3389/fpls.2024.1524163 (PMC11798799; doi:10.3389/fpls.2024.1524163)
Supplement: Supplementary file 7 [file Table6.docx]

| **Transcript name** | **Intron position** | **Splice sites** |
| --- | --- | --- |
| *CaRS31a* Isoform1 | 1^th^ | GG-GT |
| *CaRS31a* Isoform1 | 1^th^ | AG-AT |
| *CaRS31a* Isoform1 | 2^th^ | AG-GT |
| *CaRS31a* Isoform 2 | 2^th^ | AA-GG |
| *CaRS40* Isoform1 | 1^th^ | TG-CA |
| *CaRS40* Isoform1 | 1^th^ | AT-GA |
| *CaRSZ22a* Isoform1 | 1^th^ | AG-AA |
| *CaRSZ22a* Isoform1 | 3^th^ | CG-GG |
| *CaRSZ22a* Isoform 2 | 1^th^ | AG-GT |
| *CaRSZ22a* Isoform 2 | 2^th^ | CG-GG |
| *CaSR45a-1* Isoform1 | 1^th^ | AG-GG |
| *CaSR45a-1* Isoform1 | 2^th^ | AG-TG、AG-TG |
| *CaSR45a-1* Isoform1 | 3^th^ | AG-TA、AG-TC |
| *CaSR45a-1* Isoform1 | 4^th^ | TG-TA |
| *CaSR45a-1* Isoform 2 | 3^th^ | AG-AA、TG-TC |
| *CaSR45a-1* Isoform 2 | 4^th^ | TG-GT |
| *CaSR45a-1* Isoform 3 | 1^th^ | TA-GT |
| *CaSR45a-1* Isoform 3 | 4^th^ | TG-GT |
| *CaSR45a-1* Isoform 4 | 4^th^ | TG-GT |

**Supplementary table 6: Splicing sites of CaRS31a, CaRS40, CaRSZ22a, and CaSR45a-1 alternative splicing variants**
